# Mapping the TCR–Peptide-HLA Interface through Hydrogen–Deuterium Exchange Mass Spectrometry

**DOI:** 10.1021/acs.analchem.5c07287

**Published:** 2026-08-29

**Authors:** Thomas Powell, Alice Colyer, Gemma Wildsmith, Vijaykumar Karuppiah, Saher Afshan Shaikh, Martin Ebner, Andrew Creese, Antonio N. Calabrese

**Affiliations:** † Immunocore Limited, 92 Park Drive, Milton Park, Abingdon OX14 4RY, United Kingdom; ‡ Astbury Centre for Structural Molecular Biology, School of Molecular and Cellular Biology, Faculty of Biological Sciences, 70537 University of Leeds, Leeds LS2 9JT, United Kingdom

## Abstract

T-cell receptor (TCR)–based therapeutics recognize specific peptides presented by human leukocyte antigens (HLAs) on antigen-presenting cells. The docking orientation of TCRs to peptide–HLA (pHLA) complexes is critical for T-cell activation as it underpins signaling and downstream immune responses. Traditionally, TCR binding geometries with pHLAs have been resolved through X-ray crystallography. Here, we demonstrate that conventional peptide-level hydrogen–deuterium exchange mass spectrometry (HDX-MS) workflows can identify productive TCR engagement with their cognate pHLAs. By quantifying differential deuterium uptake upon complex formation, we show that TCRs that bind centrally within the pHLA groove have distinct uptake profiles compared with those adopting alternative orientations. Comparison of uptake data with information from structure prediction further contextualized the HDX data for distinct TCR–pHLA binding modes, thus highlighting the value of integrating experimental and computational approaches. Collectively, our findings establish existing HDX-MS workflows as rapid and accessible approaches for probing TCR–pHLA interactions, with direct implications for the development of TCR-based biotherapeutics.

## Graphical abstract

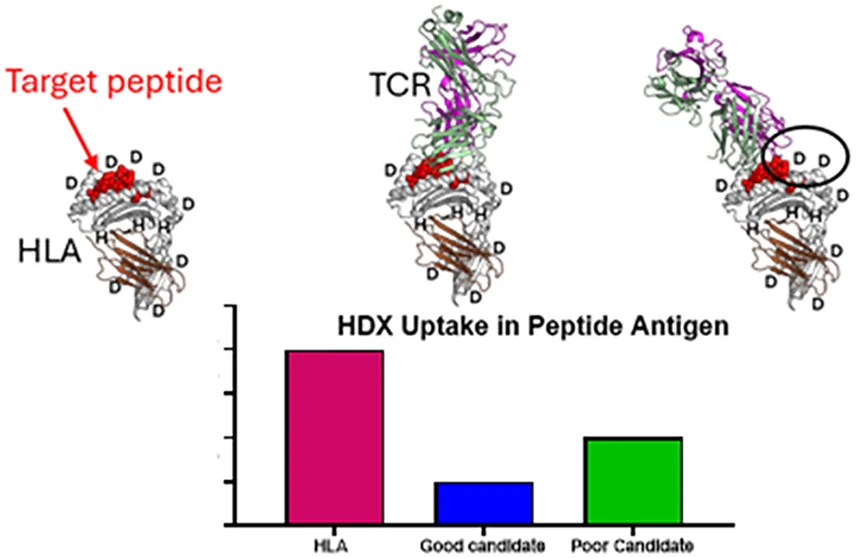

## Introduction

T-cell receptor (TCR)-based therapeutics are an innovative class of treatments that exploit the natural ability of TCRs to recognize peptide antigens presented by human leukocyte antigen (HLA) molecules on the surface of infected or malignant cells. These peptides, typically 8–13 amino acids in length, are recognized with remarkable specificity, making TCRs an attractive platform for biotherapeutic development, particularly in the realm of cancer immunotherapy.^1,2^ Class I peptide–HLA (pHLA) molecules share a conserved architecture, comprising a membrane-anchored heavy chain with three extracellular domains (α1−α3) and a nonpolymorphic β2-microglobulin (β2m) light chain.^3,4^ The α1 and α2 domains form a peptide-binding groove, consisting of a β-sheet floor flanked by two α-helices, which together accommodate the antigenic peptide.

The orientation of a TCR relative to its pHLA is a critical determinant of recognition and signaling. Structural studies reveal a highly conserved docking mode across most TCR–pHLA complexes, positioning all six complementarity-determining regions (CDRs) to engage both the peptide and the HLA surface.^5^ This geometry underpins specificity and affinity.^6^ Nonetheless, variations in docking geometry have been reported.^7,8^ For example, TCRs may shift toward the N- or C-terminus of the peptide rather than binding centrally, and substantial deviations from the peptide centroid can impair specificity.^9–11^ Even more striking are reversed docking modes, in which the TCR α- and β-chains engage opposite HLA helices; such orientations are generally less effective at eliciting immune responses.^12^ Accordingly, TCRs that adopt canonical, centrally focused binding modes are generally preferred as therapeutic candidates, but are difficult to identify from a large candidate pool.^13–15^

Given the importance of maintaining a canonical docking geometry, structural verification becomes an essential step in evaluating TCR-based therapeutic candidates. X-ray crystallography provides a direct means of confirming whether a lead molecule engages its target with the intended orientation and contact pattern.^13–15^ By resolving the three-dimensional structure of the TCR–pHLA complex at atomic resolution, crystallography reveals the precise arrangement of CDR loops over the peptide–HLA surface and highlights key interactions that define specificity and affinity. These data not only validate that a candidate adopts the desired binding conformation but also uncover unanticipated contacts or deviations that may affect potency or selectivity. Such structural confirmation is a cornerstone of structure-guided design, enabling iterative refinement and reducing the likelihood of advancing molecules with suboptimal binding profiles.

Figure 1 illustrates representative TCR–pHLA complexes analyzed in this study. TCRs from PDB 6R2L (NYB-awtb1/NYB-a1b1 variants) and PDB 9GV6 (TCR-C) exemplify canonical central binders, whereas PDB 9GV7 (TCR-E) demonstrates a reversed, noncanonical binding mode shifted toward the peptide antigen N-terminus.

**Figure 1:**
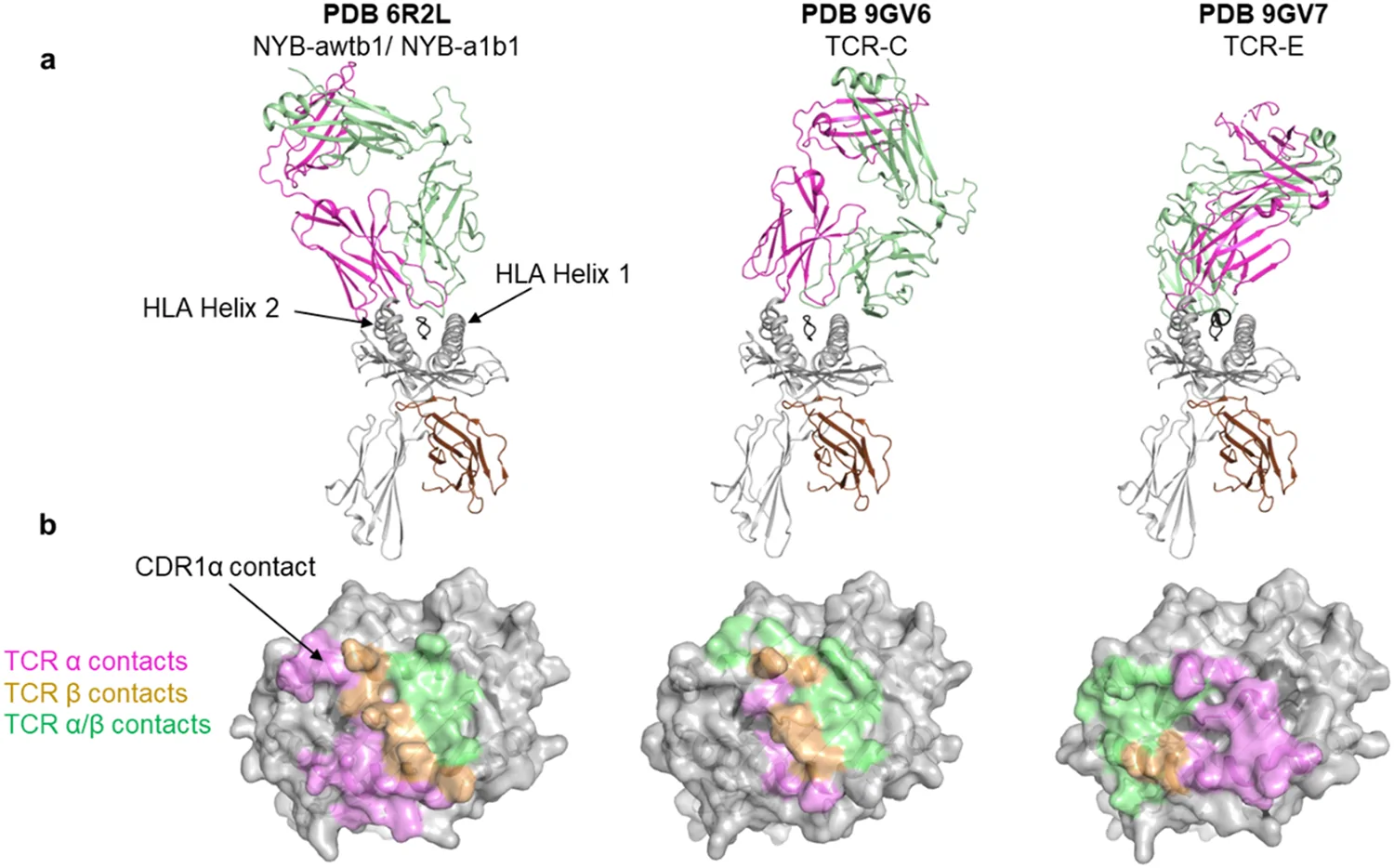
TCR–pHLA complexes can adopt distinct binding modes. (a) Crystal structures of three TCR–pHLA complexes; TCRs from PDB 6R2L (NYB-awtb1/NYB-a1b1) and 9GV6 (TCR-C) adopt canonical binding orientations. PDB 9GV7 (TCR-E) displays a reverse-binding geometry. Color scheme: TCR α chains in magenta, TCR β chains in green, peptide antigen in black, HLA in gray, and β2-microglobulin in brown. (b) Top view of the HLA surface with TCR α and β residues that approach the HLA within 4.5 Å shown as magenta and green surface patches; residues contacted by both chains are highlighted in light orange.

However, while highly informative, crystallography is technically demanding and time-consuming and requires substantial quantities of purified material.^16^ This reliance on data from crystallography represents a bottleneck in candidate selection, particularly as the number of TCRs under investigation increases. Therefore, alternative approaches are needed to facilitate the development pipeline and aid rapid TCR selection to identify canonical, centrally focused binders.

We previously developed a cross-linking mass spectrometry (XL-MS) approach capable of distinguishing canonical from reversed or shifted binding modes.^17^ However, we were only able to utilize this technique to study affinity-matured TCRs (dissociation constants (*K*_D_ values)) in the low nanomolar range and could not identify cross-links when using TCRs with affinities of ∼100 nM. We hypothesize that this was because there is an overall lack of suitable proximal residues for cross-linking (the TCR–pHLA interface is not rich in Lys residues, and we had to use the heterobifunctional sulfo-SDA cross-linker that contains a Lys-reactive NHS ester and a diazirine, which has a preference for reaction with acidic residues). Moreover, the low efficiency of the UV-activated sulfo-SDA made it challenging to capture interactions with higher dissociation constants. Thus, we sought additional methods to inform on TCR binding modes that did not rely on the presence of proximal cross-linkable residues.

We therefore turned to hydrogen–deuterium exchange mass spectrometry (HDX-MS) as a complementary method to study TCR–pHLA interactions. HDX-MS is a widely used, powerful tool for epitope mapping, including for analysis of monoclonal antibodies and other protein-based biotherapeutics. In these contexts, the method is typically used to identify the protein domain engaged by a therapeutic antibody, with peptide-level resolution generally sufficient to delineate binding regions on the relatively large and structured antigenic surfaces.^18^ It should be noted that one key challenge of HDX-MS experiments for epitope mapping is that interpretation of protection effects observed upon complex formation can be confounded by allosteric changes in structure/dynamics distal from the interaction interface, which may also manifest as ‘protection’ from exchange. However, it has been proposed that using rapid exchange experiments (fast mixing or low exchange temperatures) can help to dissect binding epitopes from allosteric effects.^19^

Previous HDX-MS studies of T-cell receptor (TCR) recognition of peptide–HLA (pHLA) complexes have primarily examined conformational changes within the TCR complementarity-determining regions or within the HLA binding groove upon complex formation, rather than mapping features of the binding interface itself when TCRs bind in different geometries.^20,21^ The TCR–pHLA system, however, presents a distinctive analytical opportunity because the principal binding surface is a short antigenic peptide rather than a folded protein domain. We hypothesized that HDX could therefore be used to report on peptide-level perturbations that directly reflect TCR engagement, providing a means to discriminate strong from weak therapeutic candidates.

Here, we demonstrate that using a conventional HDX-MS approach, utilizing peptide-level quantification of deuterium uptake, information about the broad binding architectures of TCRs relative to their pHLA targets can be obtained, alongside information about allosteric changes in protein conformation/dynamics upon binding. We exemplify this by studying the architectures/dynamics of six different TCR-pHLA complexes. Three TCRs: -C, -E and -D, were assessed in our prior manuscript that focused on epitope prediction using XL-MS.^17^ The nomenclature of these TCRs was retained to facilitate easier comparison between the two complementary studies. We show that the pattern of differential deuterium incorporation is different between TCRs that have canonical and noncanonical binding modes. The HDX data were further contextualized using experimentally determined crystal structures and computational models, providing a framework for assessing TCR–pHLA binding modes. Together, these findings establish HDX-MS as a powerful tool for guiding TCR selection and engineering strategies in immunotherapeutic development.

## Experimental Section

### Protein Expression and Purification

Six affinity-matured TCRs and their corresponding pHLA complexes were produced, refolded, and purified according to previously established protocols.^22^ Briefly, the TCR α- and β-chains were expressed separately as inclusion bodies in *E. coli* and subsequently refolded together. Likewise, the HLA heavy chain and β2-microglobulin were expressed individually as inclusion bodies in *E. coli* and refolded in the presence of a chemically synthesized peptide (Peptide Protein Research Ltd., UK). Both TCRs and pHLAs were purified by sequential anion-exchange and size-exclusion chromatography.

### Surface Plasmon Resonance

Binding analyses were performed on a Biacore T200 instrument equipped with a CM5 sensor chip. Experiments were carried out at 25 °C in PBS supplemented with surfactant P20. Streptavidin was covalently coupled via amine reactivity, and biotinylated pHLAs were immobilized on the streptavidin-coated surface, after which residual binding sites were blocked with D-biotin. TCRs were injected at six concentrations prepared by 2-fold serial dilutions, with the highest concentration set at approximately 10-fold above the expected K_D_, at a flow rate of 10 μL/min. Multicycle kinetic analysis was used to measure low-affinity interactions (NYB-awtb1 and TCR-F), while single-cycle kinetics were used to measure medium and high affinities. Data were analyzed using Biacore Insight Evaluation software (v 5.0.18.22102) and visualized using GraphPad Prism (v 10.6.0) (see Supporting Figure 1).

### HDX-MS

HDX-MS experiments were conducted with an HDX liquid handling robot (LEAP Technologies, USA) interfaced with an Acquity M-class LC and HDX Manager (Waters Corporation, UK).^23^ Protein complexes were prepared and incubated on ice for 30 min prior to starting the HDX-MS run. A total of 5 μL of protein-containing solution containing the pHLA complex (8 μM) with/without the corresponding TCR (10 μM) was added to 95 μL of deuterated buffer (1× PBS, pH 7, 95% D2O). Samples were incubated at 4 °C for 0, 0.5, 5, or 30 min. While these time points do not span 4 orders of magnitude,^24^ they provide a range of tractable time points that could be implemented in a screening/development pipeline. 4 °C was chosen as a compromise temperature to slow the rate of hydrogen exchange, relative to experiments conducted at ambient or physiological temperatures, to be able to identify subtle changes in deuterium uptake in cases where there may be rapid exchange rates (e.g., exposed peptide antigens). The exchange reactions were quenched by taking 75 μL of the sample and adding it to 75 μL of quench buffer (1× PBS, pH 1.8, 0.1% DDM). The quenched sample (90 μL) was passed through an immobilized pepsin column (Enzymate, Waters Corporation, UK), and the resultant peptides were trapped on a VanGuard Pre-Column (Acquity UPLC BEH C18 [1.7 μm, 2.1 mm × 5 mm], Waters Corporation, UK) for 3 min at a flow rate of 30 μL.min^–1^. Separation of peptide fragments was achieved with a C18 column (Acquity UPLC BEH C18 column, 130Å, 1.7 μm, 1 mm × 100 mm, Waters Corporation, UK) eluting over a linear gradient of 0%–40% (v/v) acetonitrile (0.1% [v/v] formic acid) in H2O (0.3% [v/v] formic acid) over 7 min at 40 μL min^–1^. The eluate was infused into a Synapt G2-Si mass spectrometer (Waters Corporation, UK), operated in mobility-assisted data-independent analysis (HDMSe) mode with dynamic range extension enabled.

### HDX-MS Data Analysis

Data were analyzed using PLGS (v3.0.2) and DynamX (v3.0.0) software (Waters Corporation, UK). Restrictions for peptides in DynamX were as follows: minimum intensity = 1000, maximum sequence length = 25, minimum products per amino acid = 0.3, max ppm error = 20, file threshold = 1. Deuteros 2.0 was used to identify statistically significant protected and deprotected peptides and to generate Woods plots.^25^ Statistically significant peptides were identified using a 98% confidence interval. A summary table of HDX-MS parameters, in accordance with community guidelines,^24^ can be found in Supporting Table 1 and an HDX data table can be found as a Supporting Data File.

### Structure Prediction of TCR–pHLA Complexes

Structures were generated using TCRmodel2 (TCR-D and -F) and AlphaLink2 (TCR-D), applying the programs in their published, unmodified forms.^26,27^ The XL-MS restraints used for TCR-D were as explained before.^17^ The models for NYB-awtb1 and NYB-a1b1 TCR–pHLA complexes were generated using PDB 6R2L as a template in homology modeling in Schrodinger Bioluminate (v 6.0). Buried surface area calculations were performed using the Protein Interfaces, Surfaces, and Assemblies (PISA) program.^28^ Solvent accessible surface area (SASA) for individual residues was computed using the FreeSASA program.^29^

## Results and Discussion

### Canonical TCRs with Centrally Focused Footprints Show Similar Regions of HDX-MS Protection

We first analyzed two TCR variants that target the same peptide antigen (NYB-awtb1 and NYB-a1b1; see Figure 1 and Table 1). Both exhibit centrally focused footprints on the HLA that span the full length of the antigenic peptide and engage in canonical binding modes. These variants differ by three amino acid residues in the CDR1α loop, leading to subtle alterations in their interactions with the antigenic peptide and HLA helix 2. Each buries a comparable surface area on the target peptide (356.7 for NYB-awtb1 and 322.8 Å^2^ for NYB-a1b1); however, NYB-a1b1 binds with a higher affinity. Structural models of the TCR–pHLA complexes were generated using coordinates from a previously reported structure (PDB 6R2L), but introducing appropriate single-residue changes.^30^

**Table 1:** TCRs Analyzed in This Study^a^ ^,^ ^b^

			BSA (Å^2^)		
TCR	pHLA Affinity (K_D_, nM)	Structure model and description	Peptide antigen	HLA Helix 1	HLA Helix 2
NYB-awtb1	119	Modeled mutations on crystal structure (PDB 6R2L) (Canonical, centrally focused)	356.7*	330.0*	582.6*
NYB-a1b1	0.30	Modeled mutations on crystal structure (PDB 6R2L) (Canonical, centrally focused)	322.8*	371.3*	534.7*
TCR-C	0.28	Crystal structure (PDB 9GV6) (Canonical, centrally focused)	351.2^†^	462.5^†^	204.5^†^
TCR-E	0.11	Crystal structure (PDB 9GV7) (Reverse, noncentrally focused)	211.3^†^	295.0^†^	610.7^†^
TCR-D	2.7	TCRmodel2	382.4^‡^	231.1^‡^	363.1^‡^
		Alphalink2 (incorporating XL-MS)	107.2^§^	161.6^§^	463.7^§^
TCR-F	193	TCRmodel2	246.9^‡^	298.6^‡^	206.5^‡^

a Binding affinities toward the respective pHLA complexes are reported, together with buried surface areas (BSA) on the target antigen peptide and HLA helices 1 and 2 upon TCR engagement.

b BSA values were derived from ^*^structures incorporating modeled mutations onto the crystal structure; ^†^crystal structures; ^‡^structures predicted by TCRmodel2; ^§^or structures predicted by AlphaLink2.^17^

Deuterium uptake was compared between free pHLA and TCR-bound pHLA at 0.5, 5, and 30 min of deuterium incubation (Figure 2). Subsequent peptic digestion yielded robust sequence coverage: 72.5% for the HLA heavy chain, 100% for the antigenic peptide, and 86% for β2m (Supporting Figures 2–4).

**Figure 2:**
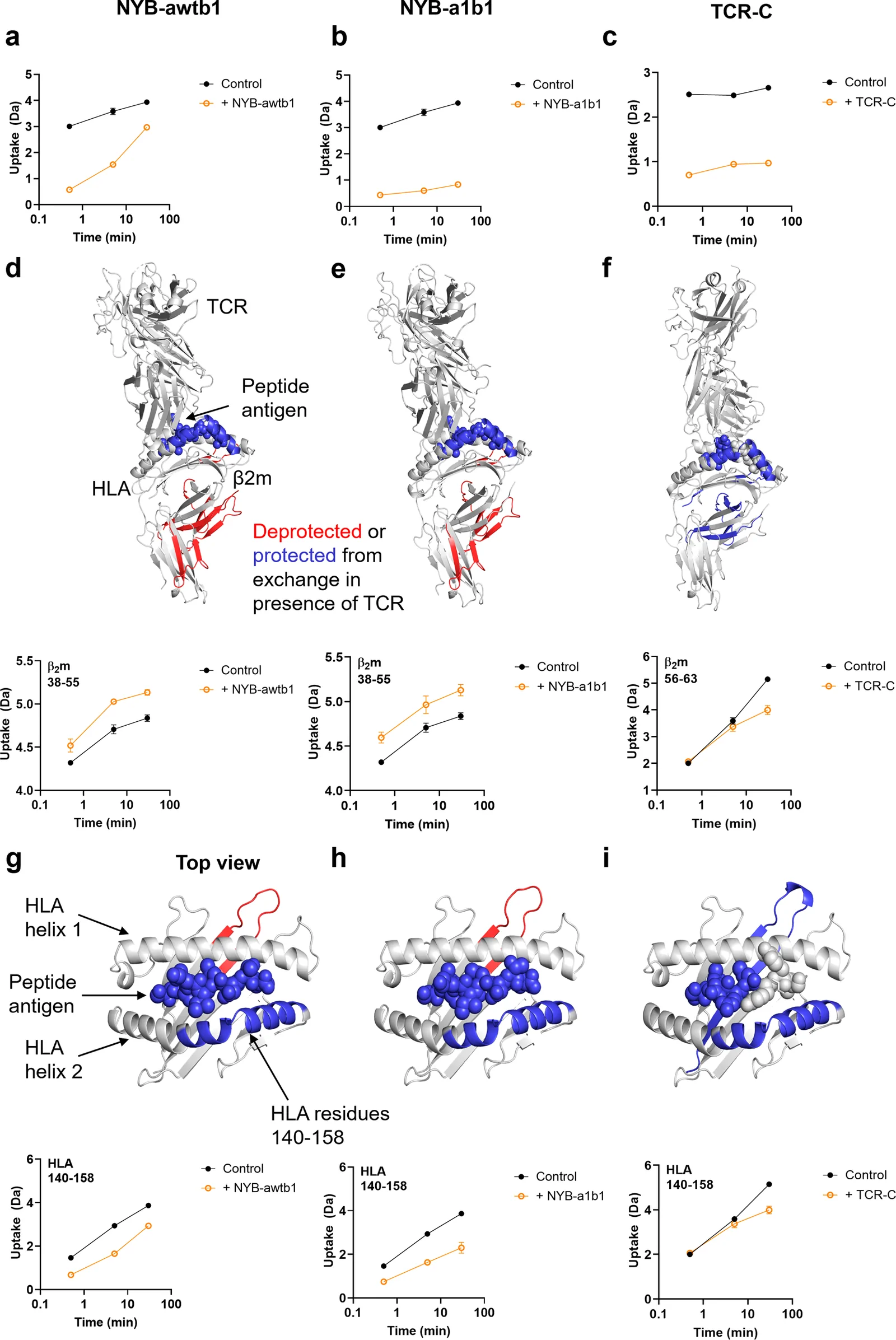
HDX analysis of TCR–pHLA complexes in canonical orientation. (a–c) Deuterium uptake plots for peptides derived from the peptide antigen of the pHLA complexes upon addition of (a) NYB-awtb1 (peptide residues 1–9), (b) NYB-a1b1 (peptide residues 1–9), and (c) TCR-C (peptide residues 1–6). Uptake at 0.5, 5, and 30 min is shown in the absence (black) or presence (orange) of the TCR. Data represent mean ± standard deviation from three independent replicates. (d–f) Mapping of significant differences in pHLA deuterium incorporation onto crystal structure or crystal structure variants of pHLA complexes in the presence of (d) NYB-awtb1, (e) NYB-a1b1, and (f) TCR-C (upper panels). Regions protected from exchange in the presence of the TCR are colored blue, and regions with increased exchange are colored red. Representative uptake plots showing regions of differential deuterium uptake in the β2m domain of the pHLA complex in the presence of the TCR (lower panels). (g–i) “Top” views of the pHLA complex when bound to (g) NYB-awtb1, (h) NYB-a1b1, and (i) TCR-C, highlighting regions with altered deuterium uptake (upper panels). Representative uptake plots showing regions of differential deuterium uptake in the HLA surrounding the peptide antigen (residues 140–158) in the presence of the corresponding TCR (lower panels). Significant differences were determined using a hybrid significance test with a 98% confidence interval, as implemented in Deuteros 2.0.^25^

In both complexes, addition of the TCR resulted in clear protection from deuterium exchange within the peptide antigen (Supporting Figures 3e and 4e), consistent with the binding footprints observed in the corresponding crystal structures. Figure 2a,b shows deuterium uptake plots of peptic peptides derived from the peptide antigen presented on the pHLA complex, with and without addition of NYB-awtb1 or NYB-a1b1. When incubated with NYB-awtb1, protection in the antigenic peptide was observed over all time points, but the extent of the protection decreased with increased labeling time. This indicates conformational flexibility in the bound state, suggesting that exchange-competent conformations are populated or that the complex has a relatively rapid dissociation rate. Conversely, the extent of uptake in the NYB-a1b1 bound state was consistent over all incubation time points, suggesting a slower off-rate and/or reduced conformational dynamics. This observation is consistent with the higher binding affinity of NYB-a1b1 (*K*_D_ = 0.30 nM) compared to NYB-awtb1 (*K*_D_ = 119 nM) and demonstrates that analysis of exchange kinetics in HDX-MS deuterium uptake plots can indicate relative TCR binding affinities.

TCR-C is another example of a TCR that adopts a canonical, centrally focused binding mode. The buried surface area of the antigenic peptide upon TCR-C engagement, as calculated from the crystal structure, was comparable to that observed when NYB-awtb1 and NYB-a1b1 bound their cognate pHLA complexes (351.2 Å^2^). Deuterium uptake was assessed between free pHLA and the TCR-C-pHLA complex. Again, addition of TCR-C induced a marked reduction in deuterium uptake within the peptide antigen relative to pHLA alone (Figure 2c and Supporting Figure 2, 5). The extent of deuterium uptake was consistent over all incubation time points, as observed for the NYB-a1b1-pHLA complex, suggesting a similarly slow off-rate and/or reduced conformational dynamics in the bound state, in line with the high binding affinity of TCR-C (*K*_D_ = 0.28 nM).

Given their similar interaction networks and binding orientations, these complexes are expected to display broadly comparable regions of protection across both HLA and peptide in the TCR-bound state. Interestingly, distal effects were also evident, with changes in deuterium uptake detected in the β2m domain in the bound state (Figure 2d–f). This observation is consistent with the ability of HDX-MS to report on allosteric changes in structure/dynamics during epitope mapping experiments and with previous reports that β2m can modulate TCR–pHLA recognition kinetics.^21,31^

In summary, for each of these complexes tested, when each TCR was added to its cognate pHLA, deuterium uptake was largely reduced for the antigenic peptide. This agrees with the crystal structures and calculated BSAs (Table 1). Protection was also observed around the peptide-binding crevice of the HLA heavy chain, particularly at helix 2 residues spanning ∼140–158 (Figure 2g–**i**).

### HDX-MS Reveals Altered Protection in a Reversed-Orientation TCR

We previously published the crystal structure of TCR-E (PDB: 9GV7), which revealed a noncanonical binding footprint relative to its cognate pHLA. In this structure, the TCR adopts a reversed orientation, with the α- and β-chains interchanged at the interface (Figure 1). To assess this binding mode by HDX-MS, deuterium uptake was compared between free pHLA and the TCR-E-pHLA complex (Supporting Figures 2, and 6). In contrast to the centrally focused binders, addition of TCR-E resulted in increased deuterium uptake within the peptide antigen (Figure 3a–d). This was unexpected, as the crystal structure shows TCR-E positioned above a substantial portion of the peptide despite its shifted footprint (BSA of 211 Å^2^). Notably, no protection was observed around the HLA binding crevice, a region protected in the canonical binders (Figures 2g–i and 3d).

**Figure 3:**
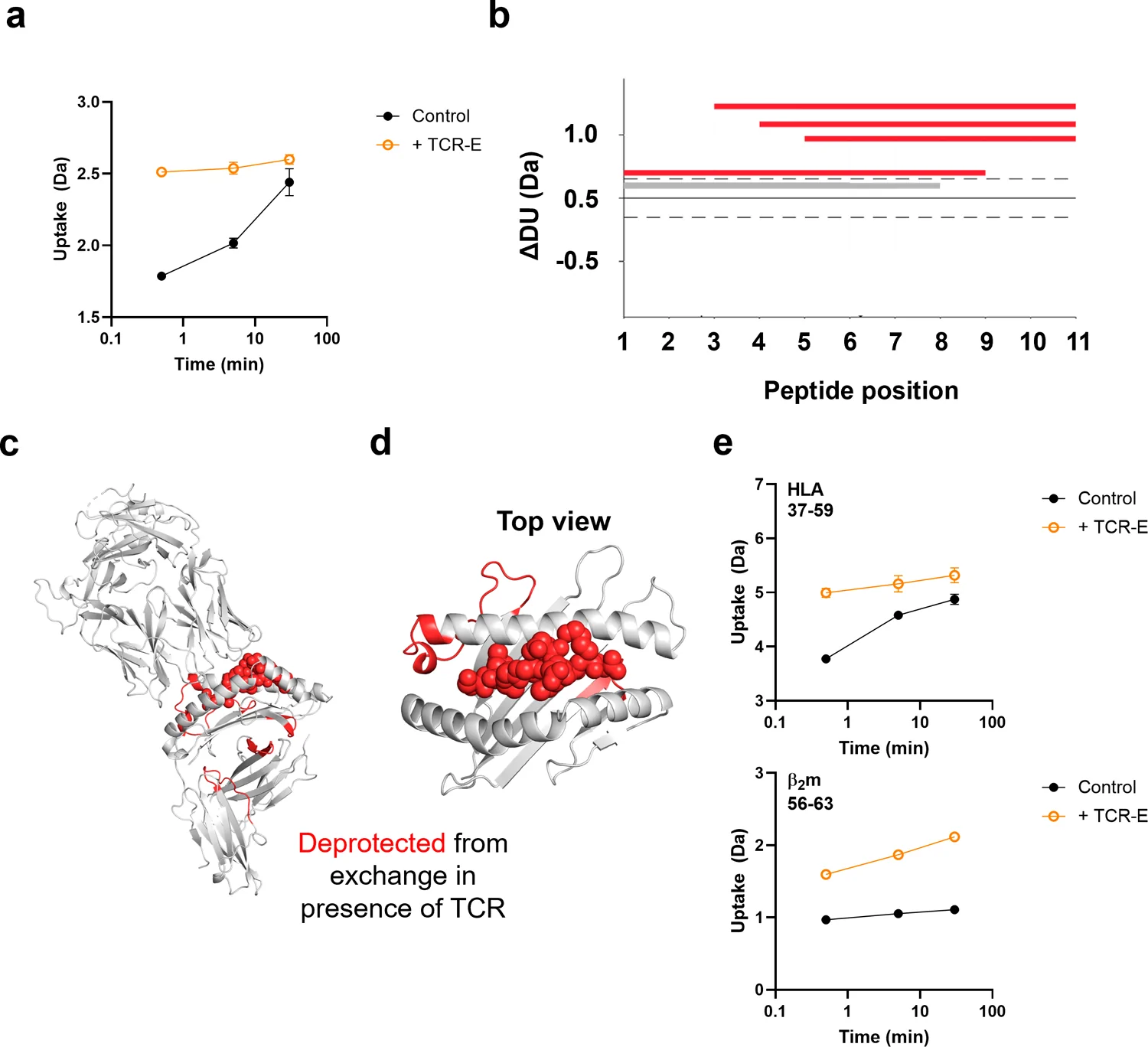
HDX analysis of the TCR-E-pHLA complex, which binds in a noncanonical orientation. (a) Deuterium uptake plots for a peptide derived from the peptide antigen (peptide residues 3–11) of the pHLA complex upon binding to TCR-E. Uptake is shown at 0.5, 5, and 30 min in the absence (black) or presence (orange) of TCR-E. Data represent mean ± standard deviation from three independent replicates. (b) Woods’ plot showing differences in deuterium incorporation at the 30 s time point for the peptide antigen. (c) Mapping of significant differences in pHLA deuterium incorporation onto the crystal structure of the TCR-E-pHLA complex. Regions exhibiting increased exchange upon TCR binding are highlighted in red. (d) “Top” view of the HLA binding groove, emphasizing regions with altered deuterium uptake. (e) Representative uptake plots showing regions of differential deuterium uptake in the HLA (upper panel) and β2m domain (lower panel) in the presence of TCR-E.

TCR-E recognizes the 11-mer antigenic peptide (SLAGGLDDMKA), whose crystal structure reveals a pronounced twist between residues 5–9 to accommodate binding within the HLA groove. We hypothesize that this conformation represents an induced fit driven by the TCR, whereas in the unbound state the peptide likely adopts a more extended orientation, partially protruding from the HLA groove. Consistent with this hypothesis, in the absence of TCR-E we observe increased deuterium incorporation with incubation time in the peptide antigen, suggesting conformational flexibility, whereas in the presence of TCR-E the extent of deuterium incorporation does not increase over time, suggesting a slower off-rate of the complex or a less dynamic structure. Such conformational differences in the unbound state could explain the differences in deuterium uptake observed by HDX-MS in the presence of TCR-E, as solvent accessibility is markedly altered between bound and unbound states. Such changes in hydrogen exchange could additionally result from allosteric effects unrelated to epitope binding. Understanding the molecular basis of this observation would require computational simulations or other additional experiments which are beyond the scope of this study. Nevertheless, these data demonstrate that reverse binders might have different deuterium exchange profiles to canonical central binders.

Additional regions of deprotection were also detected across the HLA heavy chain and in the β2m domain (Figure 3c–e), distinct from the patterns observed with the canonical binders previously discussed. Interestingly, deuterium uptake in the peptide antigen remained comparable across all three time points in the presence of TCR-E, a trend also observed for NYB-a1b1 and TCR-C. This may reflect the very high affinity of TCR-E (*K*_D_ = 0.11 nM). Taken together, the distinct HDX-MS fingerprint of this TCR that binds in a noncanonical orientation, compared with canonical, centrally focused binders, highlights the potential of HDX-MS to identify TCRs with atypical binding modes and demonstrates the potential of HDX-MS as a screening tool during TCR development.

### HDX-MS of TCRs with Unresolved Binding Modes

To extend our analysis, two TCRs (TCR-D and TCR-F) of unknown binding footprints were assessed using HDX-MS (Figure 4). Both receptors recognize the same peptide antigen, but crystal structures of these complexes have not yet been resolved. Notably, TCR-D was previously characterized by XL-MS in our earlier study.^17^ Our aim was to evaluate whether HDX-MS can serve as a screening tool to identify TCRs that are optimally positioned relative to their cognate pHLA, thereby informing candidate selection for therapeutic development. Each TCR was analyzed as described above, with coverage maps and Wood’s plots provided in Supporting Figures 7–8.

**Figure 4:**
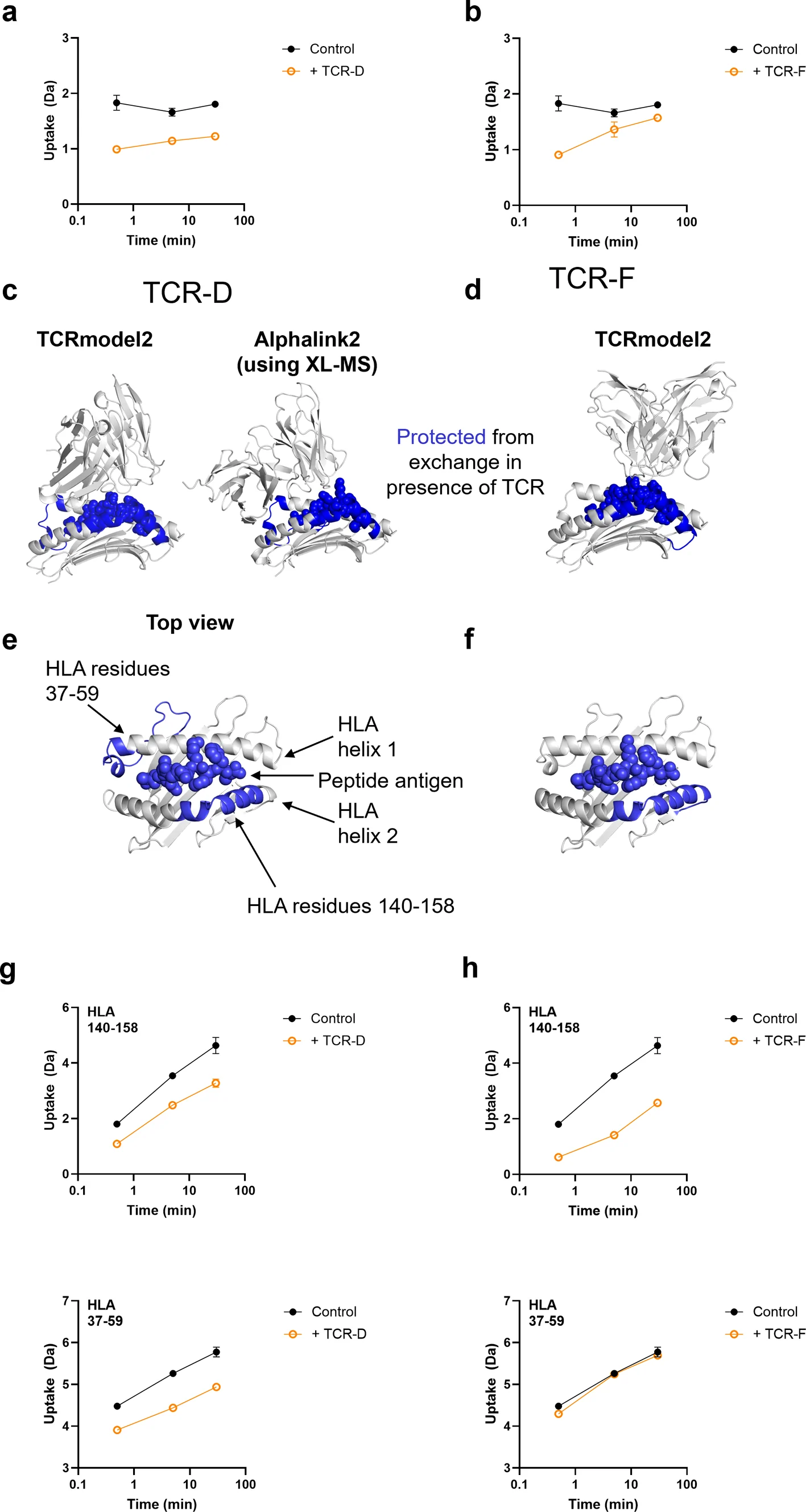
Comparison of HDX data for TCRs with unknown binding modes. (a, b) Deuterium uptake plots for peptides derived from the peptide antigen (peptide residues 1–9) from the pHLA complex upon binding of (a) TCR-D and (b) TCR-F. Uptake is shown at 0.5, 5, and 30 min in the absence (black) or presence (orange) of the respective TCR. Data represent mean ± standard deviation from three independent replicates. (c) Structural mapping of significant differences in pHLA deuterium incorporation onto the TCR-D–pHLA complex predicted with TCRmodel2 (left) or Alphalink2 (right; incorporating experimental XL-MS restraints). (d) Mapping of pHLA significant differences for the TCR-F-pHLA complex using the TCRmodel2-predicted structure. (e, f) Top views of the HLA binding groove highlighting regions with altered deuterium uptake. (g, h) Representative uptake plots showing regions of differential deuterium uptake in the HLA in the presence of (g) TCR-D or (h) TCR-F, corresponding to the regions highlighted in panels e,f (residues 140–158, upper panels, and 37–59, lower panels).

Figure 4a,b present deuterium uptake plots for peptides derived from the pHLA in the presence and absence of TCR-D and TCR-F. In comparison to the centrally focused canonical binders, TCR-D resulted in only modest protection of the antigenic peptide, suggesting a suboptimal binding mode. The extent of uptake did not change over time, suggesting a slow off-rate or restrained dynamics. Uniquely, we also observed protection from exchange in the HLA at residues 37–59, which is in a region that is adjacent to the antigenic peptide (Figure 4e,g). Protection in this region was not seen in any of the canonical binders with centrally focused footprints studied (Figure 2).

Comparable results were obtained for TCR-F, which also exhibited reduced protection in the antigenic peptide relative to the canonical binders. Moreover, deuterium uptake kinetics of the peptide antigen in the bound state were consistent with a fast off-rate/conformational flexibility (increased uptake over time). This trend correlates with binding affinities, with TCR-D (*K*_D_ = 2.7 nM) binding more tightly than TCR-F (*K*_D_ = 193 nM). No protection was observed at residues 37–59 of the HLA when TCR-F was added (Figure 4f,h).

### Integrating HDX-MS with Structural Prediction of TCR–pHLA Complexes

To further interpret these findings, structural prediction of pHLA complexes with TCR-D and TCR-F was initiated using TCRmodel2.^26^ This tool leverages the AlphaFold2 deep-learning framework,^32^ with modifications for multiple sequence alignments and template selection. TCRmodel2 plots the position of the binding regions of the TCR α and β chains against the HLA peptide-binding groove. Interestingly, TCRmodel2-predicted TCR-D to bind centrally (Figure 4c), providing a BSA for the target peptide antigen of 382.4 Å^2^. However, upon binding of TCR-D, we only observed weak protection of the peptide antigen from exchange when compared to other canonical binders (<1 Da difference in uptake between the bound/unbound states; compare Figure 4a with Figure 2b,c). This suggests altered docking of the TCR-D to the pHLA complex. This discrepancy highlights the limitations of deep learning-based structural predictions when applied to noncanonical docking geometries and demonstrates that complementary orthogonal experimental validation of structural models is required. In our previous work, we demonstrated that integrating cross-linking mass spectrometry (XL-MS) with computational modeling improves prediction of noncanonical binding modes.^17^ We have previously shown that remodeling the TCR-D complex with XL-MS restraints (Figure 4c) positioned TCR-D away from the peptide centroid and shifted toward the N-terminus (BSA recalculated as 107.2 Å^2^).

As previously mentioned, we also observed a further region of protection in the HLA upon TCR-D binding, adjacent to the peptide-binding crevice at residues 37–59 in HLA helix 1 (Figure 4e,g) that we do not observe in any canonical, centrally focused binders (Figure 2). To allow for easy comparison between the different data sets presented here, we plotted the difference in deuterium uptake at each incubation point for a peptide spanning residues 37–59 in the HLA across the canonical binding TCRs tested (Figure 5a). This region of the HLA binding crevice is uniquely protected from deuterium exchange in TCR-D. This correlates with the modeled TCR-D-pHLA complex which, when integrated with XL-MS data, shows the binding footprint of TCR-D shifted toward the N-terminus of the peptide antigen (Figure 4c).^17^ To demonstrate this concordance of our HDX-MS data with the structural model from TCRmodel2, we calculated solvent accessibility changes observed in the pHLA, in the presence of TCR-D on a per-residue basis (Figure 5b). It is clearly visible that the binding footprint of the TCR in this complex is shifted toward the peptide N-terminus compared with centrally focused TCRs (Figure 5c and Supporting Figure 9), with solvent accessibility changes in the HLA surrounding the peptide also shifted toward the peptide N-terminus. Protection from exchange in this region (residues 37–59) of the HLA could therefore be diagnostic for binders whose epitope is shifted toward the peptide N-terminus.

**Figure 5:**
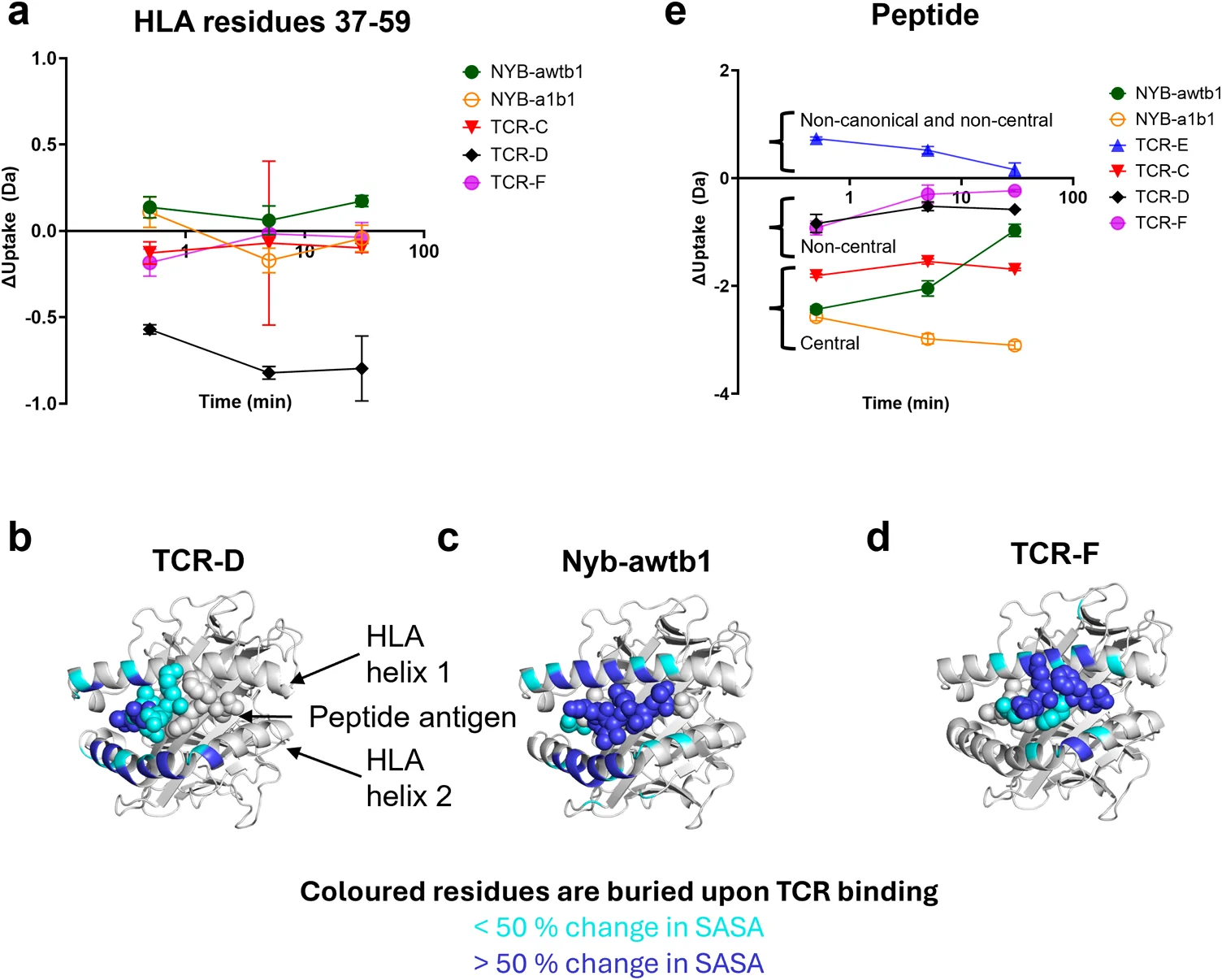
Differential deuterium uptake upon TCR binding. (a) Peptides spanning HLA residues 37–59 were analyzed for differential deuterium uptake in the presence of TCRs at 0.5, 5, and 30 min. (b, c, d) Solvent accessibility for each residue was calculated in the presence/absence of the TCR. Residues with decreased solvent accessibility in the pHLA when its partner TCR is added. (b) TCR-D, (c) pHLA-Nyb-awtb1, and (d) TCR-F are shown in light blue (<50% change in SASA) or dark blue (>50% change in SASA). (e) Peptides spanning the peptide antigen were analyzed for differential deuterium uptake in the presence of TCRs at 0.5, 5, and 30 min. Data represent mean ± standard deviation from three independent replicates.

For TCR-F, the top-ranked model unrealistically placed the TCR beneath the binding groove and was discarded. The second-ranked model predicted a shifted orientation toward the peptide antigen C-terminus (Figure 4d). Based on the TCRmodel2 structure, the BSA for the target peptide antigen in the presence of TCR-F was calculated as 246.9 Å^2^, less than the tested canonical binders (Table 1). This prediction of a nonoptimal binding geometry aligns with our observation of reduced protection in the peptide antigen compared with canonical binders (compare Figure 4b, Figure 2a–c). Additionally, no protection was observed in HLA residues 37–59, consistent with the TCR predicted to be positioned toward the peptide antigen C-terminus (Figure 5d).

## Conclusions

In this study, we demonstrated that HDX-MS can differentiate distinct binding footprints of TCRs relative to their cognate pHLAs. To summarize, we sought to visualize all of the differential deuterium uptake data for the peptide antigens in all TCR–pHLA complexes studied (Figure 5e). This combined data set demonstrates that the canonical, centrally focused binders (NYB-awtb1, NYB-a1b1, and TCR-C) confer the strongest protection of the target peptide antigen from deuterium exchange. In contrast, TCR-E, which binds in a reversed, noncanonical orientation shifted toward the peptide N-terminus, failed to protect the peptide antigen and instead increased solvent accessibility—most notably at the C-terminal region. Similarly, TCR-D and TCR-F, with predicted shifted binding footprints, displayed reduced protection compared with centrally focused binders, consistent with suboptimal positioning. In the case of TCR-D, the binding footprint was significantly shifted such that an altered region of protection on the HLA was also observed.

HDX-MS has previously been used to map epitopes for centrally focused TCR–pHLA complexes. Merkle et al. probed the conformational dynamics of different TCR variants upon binding to their cognate pHLA.^21^ Similar to our findings, the authors showed significant protection on HLA residues surrounding the binding groove. In Merkle’s study, HLA residues 140–159 showed protection from exchange; however, additional regions of protection at residues 34–60 and 61–84 were also reported, which were not observed in our data. This suggests that while HDX consistently identifies core binding regions, differences in TCR orientation and affinity can yield distinct protection patterns. While further exploration of these distal effects lies beyond the scope of this study, they will be addressed in future work.

Other MS-based footprinting methods, including fast photochemical oxidation of proteins (FPOP) and XL-MS, offer complementary insights.^17,33^ FPOP works using a similar premise to HDX, in that it is effective at revealing regions of an antigen that become less solvent accessible upon TCR binding, thus identifying the epitope.^34^ It is important to note here that the conventional HDX-MS approach we used here was limited to peptide-level resolution, and most FPOP approaches also result in peptide-level data. Both scenarios could lead to overestimation of epitopes. For example, in TCRs NYB-awtb1, NYB-a1b1, TCR-C, and TCR-F, some residues (∼140–159) in the HLA surrounding the binding crevice may be incorrectly assigned as corresponding to binding epitopes, reflecting the limitations of mapping via peptide-resolution data. Additionally, both HDX and FPOP assess “protection” rather than direct “binding”. Consequently, any allosteric conformational changes during binding that reduce solvent exposure are also identified as being protected from exchange and could be misassigned as epitopes.^35^ Going forward, recent advances in HDX-MS analysis could be exploited to determine deuterium uptake at residue resolution, providing granular detail about modes of TCR engagement with HLAs, overcoming potential artifacts associated with peptide-level resolution HDX-MS data.^36,37^ Such single-residue resolution experiments may enable further discrimination of diverse binding modes, especially in the context of a TCR–pHLA complex, where residue-resolved deuterium uptake by the peptide antigen termini (either N- or C−) could help to identify the region of the peptide that remains exposed in the bound state.

In contrast to the HDX-MS data we present here, XL-MS provides complementary information to HDX as it measures contact points directly and can achieve amino acid resolution more easily. However, it may underestimate the epitope due to the limited reactivity of the linker used, and additional complexities in understanding the fragmentation data often lead to lengthier analysis times.^38^ In previous work studying similar complexes with XL-MS, the sensitivity of the technique often restricted analysis to later-stage affinity-matured TCRs, and valuable data could only be obtained for TCRs with *K*_D_ values in the low-nM range (<∼20 nM). By contrast, in the HDX-MS data presented here, informative footprinting was observed even for TCR-F, which had a *K*_D_ of ∼200 nM. This raises the possibility of integrating HDX-MS at earlier stages of discovery pipelines compared with XL-MS. Nevertheless, XL-MS retains unique strengths. By localizing contact sites to individual residues, it can unambiguously identify docking orientation, such as α-chain positioning over HLA helix 2 in canonical binders. While HDX-MS revealed differences between canonical and reversed complexes, it cannot empirically resolve orientation with the same precision. Thus, the two methods are best viewed as complementary.

As with our prior study, the HDX-MS data collected here highlighted the inherent risks of relying solely on predicted models without experimental validation. This is exemplified by the TCR-D-pHLA complex, which was initially predicted to bind with a broadly central footprint (Figure 4c), yet HDX-MS data indicated a nonoptimal configuration. While recent advances in deep learning–based artificial intelligence have significantly improved the accuracy of predicting therapeutic epitopes and their interactions, limitations remain. Unconventional binding modes—such as reverse docking or TCRs positioned heavily toward the peptide termini—pose challenges to current models of TCR–pHLA interactions. This underscores the necessity for predictive models to account for alternative binding modes, which are crucial for biotherapeutic design and predicting immune responses. Our study emphasizes the importance of integrating computational predictions, which may be unreliable, with experimental validation.

Here we have purposely designed our HDX-MS experimental configuration so that it is primed for use in a TCR development pipeline. For example, we deliberately did not choose excessively long or short deuterium incubation time points, which might require manual intervention or long labeling/instrument usage times (time points spanning 4 orders of magnitude are recommended). While this may mean that subtle changes in the dynamics of pHLAs in complex with TCRs could have been missed by our approach, here we show that it is possible using the time points tested to detect and monitor differences in modes of TCR engagement. We also make an intriguing observation that TCR-E binding to its pHLA complex results in increased deuterium incorporation. Elucidating the mechanistic basis of this phenomenon, for example through molecular dynamics simulations, will be essential for understanding the complex conformational dynamics that underpin TCR–pHLA recognition.

In summary, HDX-MS provides a powerful and accessible approach for probing TCR–pHLA interactions. We show that it can distinguish between canonical and noncanonical binding modes, as well as centrally focused TCRs from those that bind away from the peptide antigen centroid. Even in the absence of crystallographic data, HDX-MS can generate meaningful insights at affinities that extend beyond the practical reach of XL-MS. While the peptide-level deuterium exchange data we present here lacks residue-level resolution, its speed, simplicity, and sensitivity make it particularly well suited for early-stage screening of TCR candidates. Nevertheless, future work should explore if residue-resolution data could provide additional confidence to discriminate between different binding modes. Importantly, our study highlights the value of integrating HDX-MS with complementary methods, including XL-MS and computational modeling, to achieve a more comprehensive understanding of TCR binding geometries. In this context, while AI-based tools for predicting TCR–pHLA interactions can offer useful hypotheses, we advocate that their outputs should be interpreted with caution, and that all structural models should be validated and/or guided by experimental data. Here, we show that HDX-MS is a useful tool to provide such experimental validation. Beyond TCRs, the principles demonstrated here are broadly applicable to other protein–protein complexes of therapeutic interest, such as antibody–antigen interactions. Together, these findings support the incorporation of HDX-MS into discovery pipelines as a practical tool for guiding candidate selection and informing engineering strategies.

## Supplementary Material





## Data Availability

The HDX-MS data have been deposited to the ProteomeXchange Consortium via the PRIDE partner repository with the data set identifier PXD070888.

## Notes

HDX data table including details of all the peptides included in the study (XLSX)
Additional details of SPR and HDX-MS experiments (PDF)
