# Supplementary material for: Mapping the TCR–Peptide-HLA Interface through Hydrogen–Deuterium Exchange Mass Spectrometry
Source: Anal Chem. 2026 Aug 29;98(36):26299–310. doi: 10.1021/acs.analchem.5c07287 (PMC13584625; doi:10.1021/acs.analchem.5c07287)
Supplement: Supplementary file 1 [file ac5c07287_si_002.pdf]

Supporting Information

Mapping the TCR–Peptide-HLA Interface Through Hydrogen–Deuterium Exchange Mass Spectrometry

Thomas Powell[1]*, Alice Colyer[2], Gemma Wildsmith[2], Vijaykumar Karuppiah[1], Saher Afshan Shaikh[1], Martin Ebner[1], Andrew Creese[1] and Antonio N Calabrese[2*]

[1]Immunocore Limited, 92 Park Drive, Milton Park, Abingdon, OX14 4RY, United Kingdom

[2]Astbury Centre for Structural Molecular Biology, School of Molecular and Cellular Biology, Faculty of Biological Sciences, University of Leeds, Leeds, LS2 9JT, UK

* Correspondence to: thomas.powell@immunocore.com, a.calabrese@leeds.ac.uk.

Table of Contents

Supplementary Figure 1: SPR sensorgrams for TCRs tested in this study. S3

Supplementary Figure 2: Representative sequence coverage maps.......................... S4

Supplementary Figure 3: HDX-MS of the NYB-awtb1-pHLA complex......................... S5

Supplementary Figure 4: HDX-MS of the NYB-a1tb1-pHLA complex.......................... S6

Supplementary Figure 5: HDX-MS of the TCR-C-pHLA complex................................ S7

Supplementary Figure 6: HDX-MS of the TCR-E-pHLA complex................................ S8

Supplementary Figure 7: HDX-MS of the TCR-D-pHLA complex................................ S9

Supplementary Figure 8: HDX-MS of the TCR-F-pHLA complex. S10

Supplementary Figure 9: Solvent accessibility changes in pHLAs upon binding S11

Supplementary Table 1. HDX Data Summary Table. S12

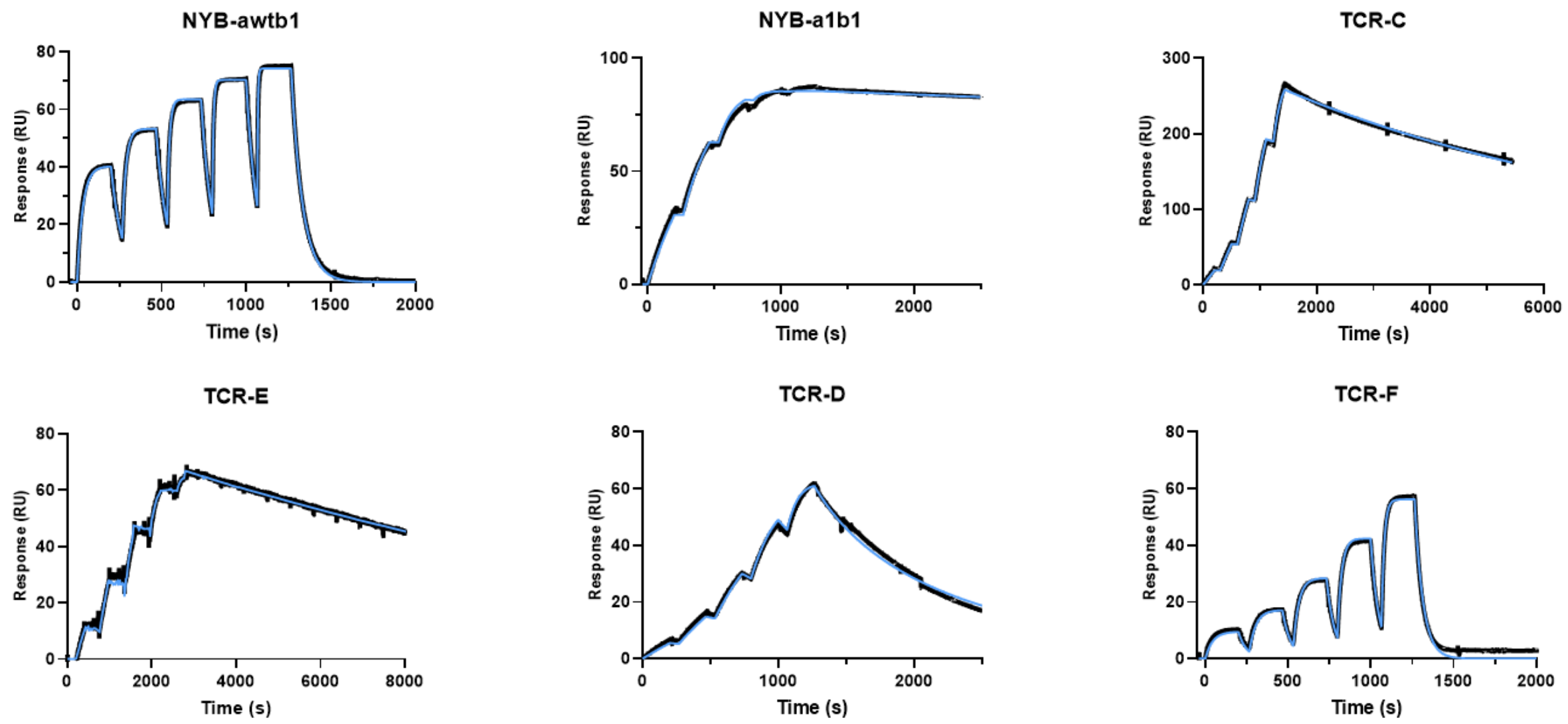

Supplementary Figure 1: SPR sensorgrams for TCRs tested in this study. The black traces represent the experimental data, and the blue traces show the corresponding global fits.

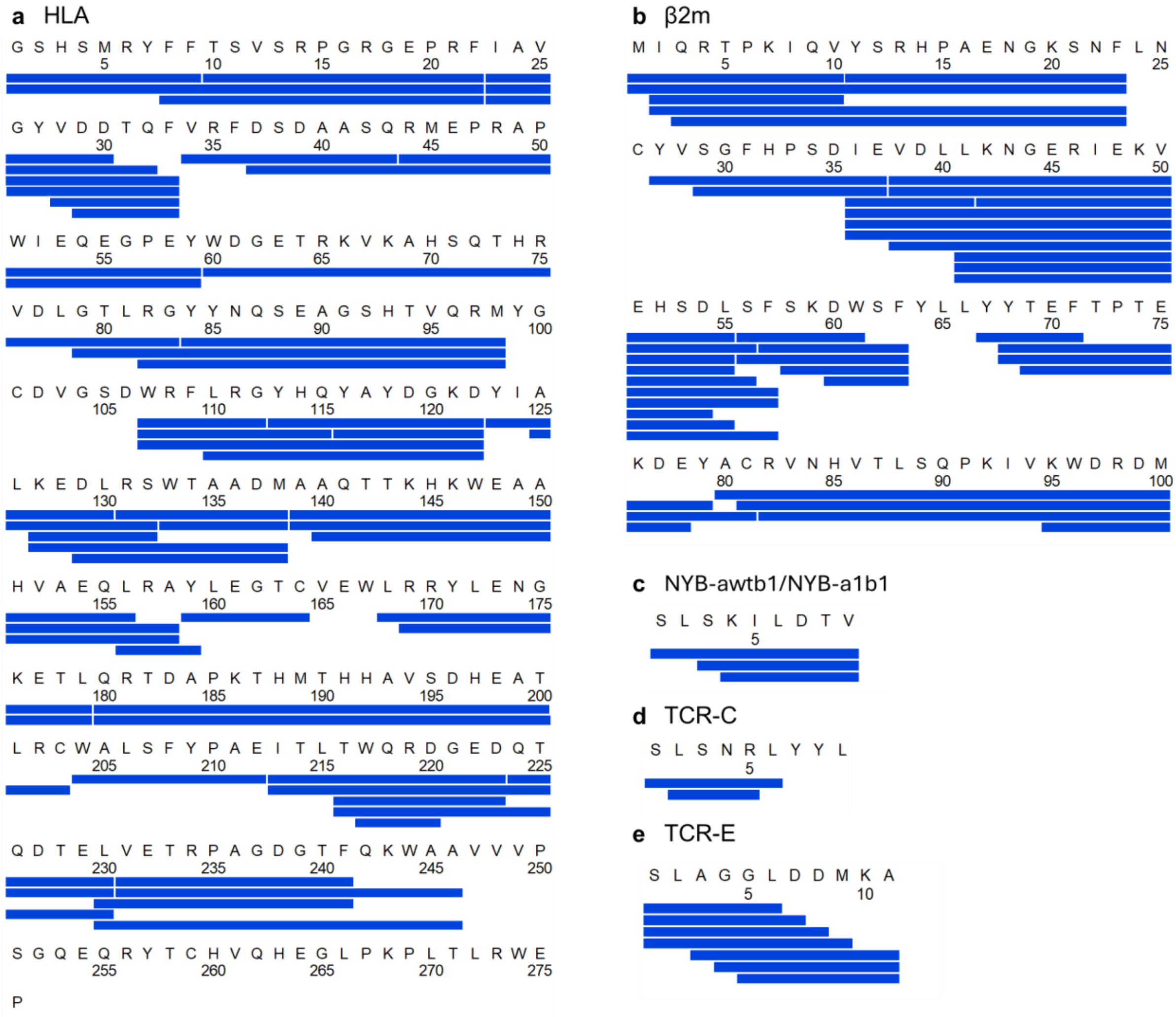

Supplementary Figure 2: Representative sequence coverage maps. Coverage and protein sequences for **a** HLA, **b** β2m and **c-e** target peptide antigens in HDX-MS experiments.

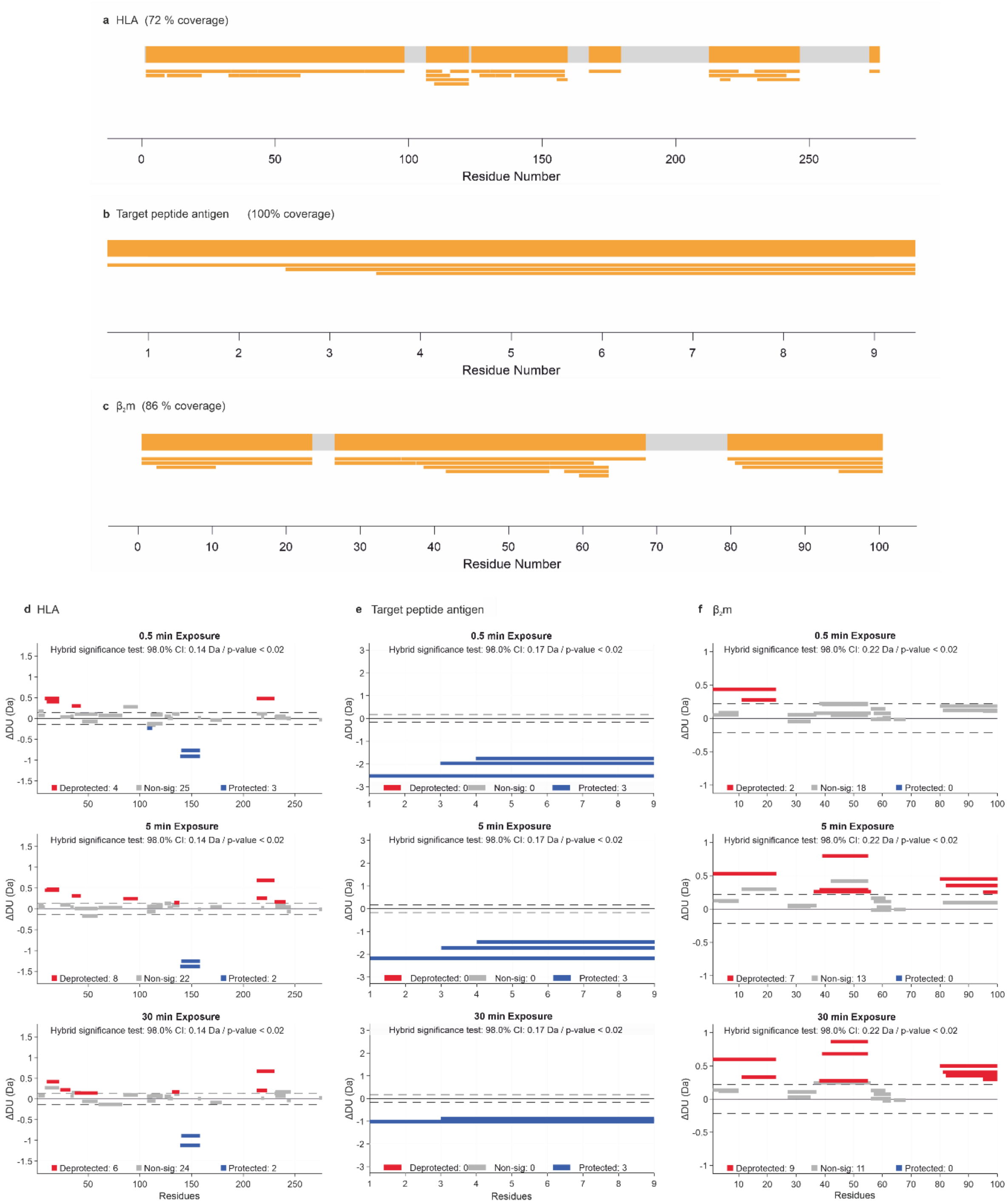

Supplementary Figure 3: HDX-MS of the NYB-awtb1-pHLA complex. Sequence coverage map of **a** HLA, **b** target peptide antigen and **c** β2m in HDX-MS experiments for NYB-awtb1. Woods' plot showing the differences in deuterium incorporation in each timepoints for **d** HLA, **e** target peptide antigen and **f** β2m domains. Peptides from the pHLA that were significantly protected and deprotected when incubated with deuterium in the presence of the TCR are shown in blue and red, respectively (hybrid statistical test, $p < 0.02$).

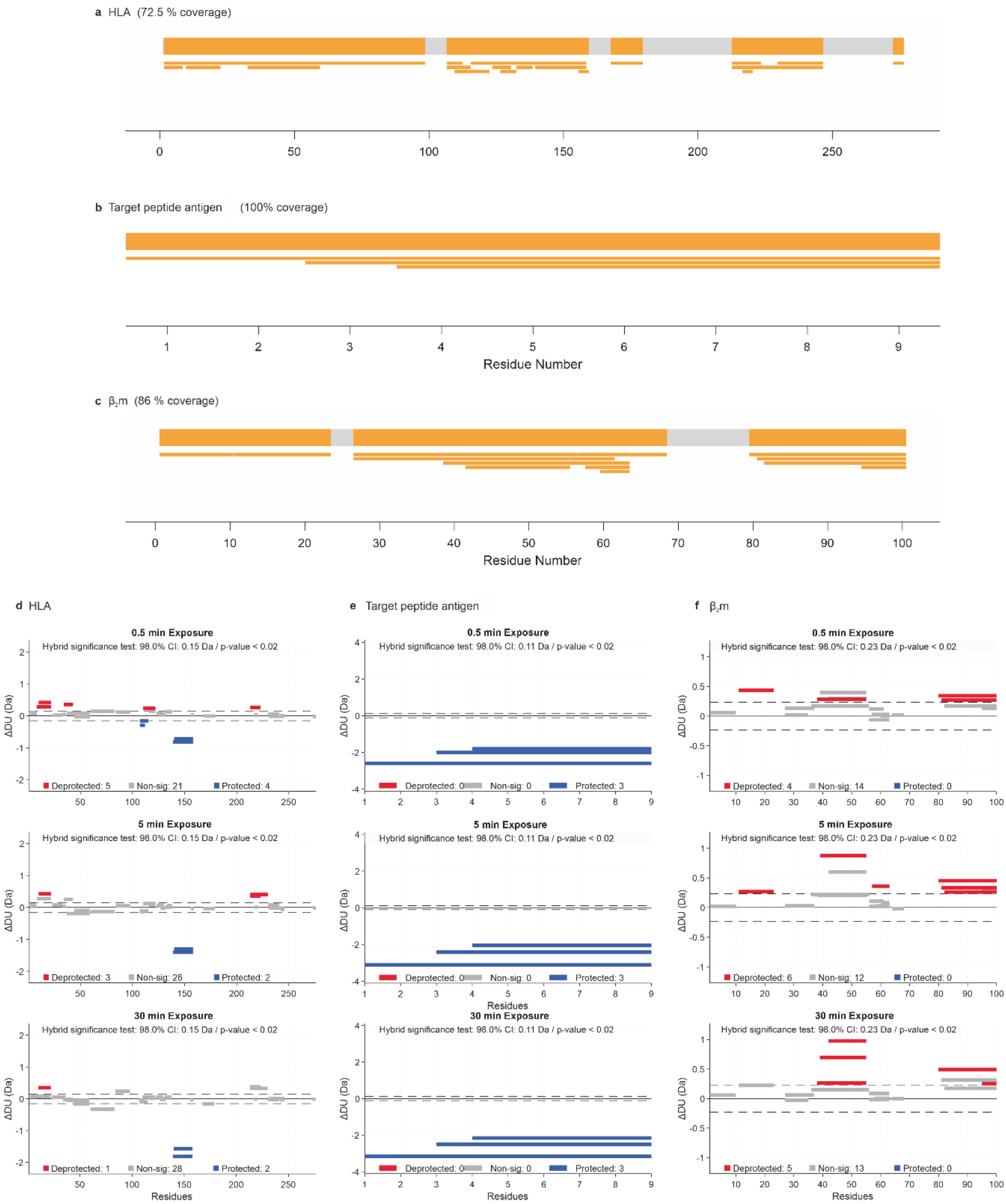

Supplementary Figure 4: HDX-MS of the NYB-a1b1-pHLA complex. Sequence coverage map of **a** HLA, **b** target peptide antigen and **c** β2m in HDX-MS experiments for NYB-a1b1. Woods' plot showing the differences in deuterium incorporation in each timepoints for **d** HLA, **e** target peptide antigen and **f** β2m domains. Peptides from the pHLA that were significantly protected and deprotected when incubated with deuterium in the presence of the TCR are shown in blue and red, respectively (hybrid statistical test, $p < 0.02$).

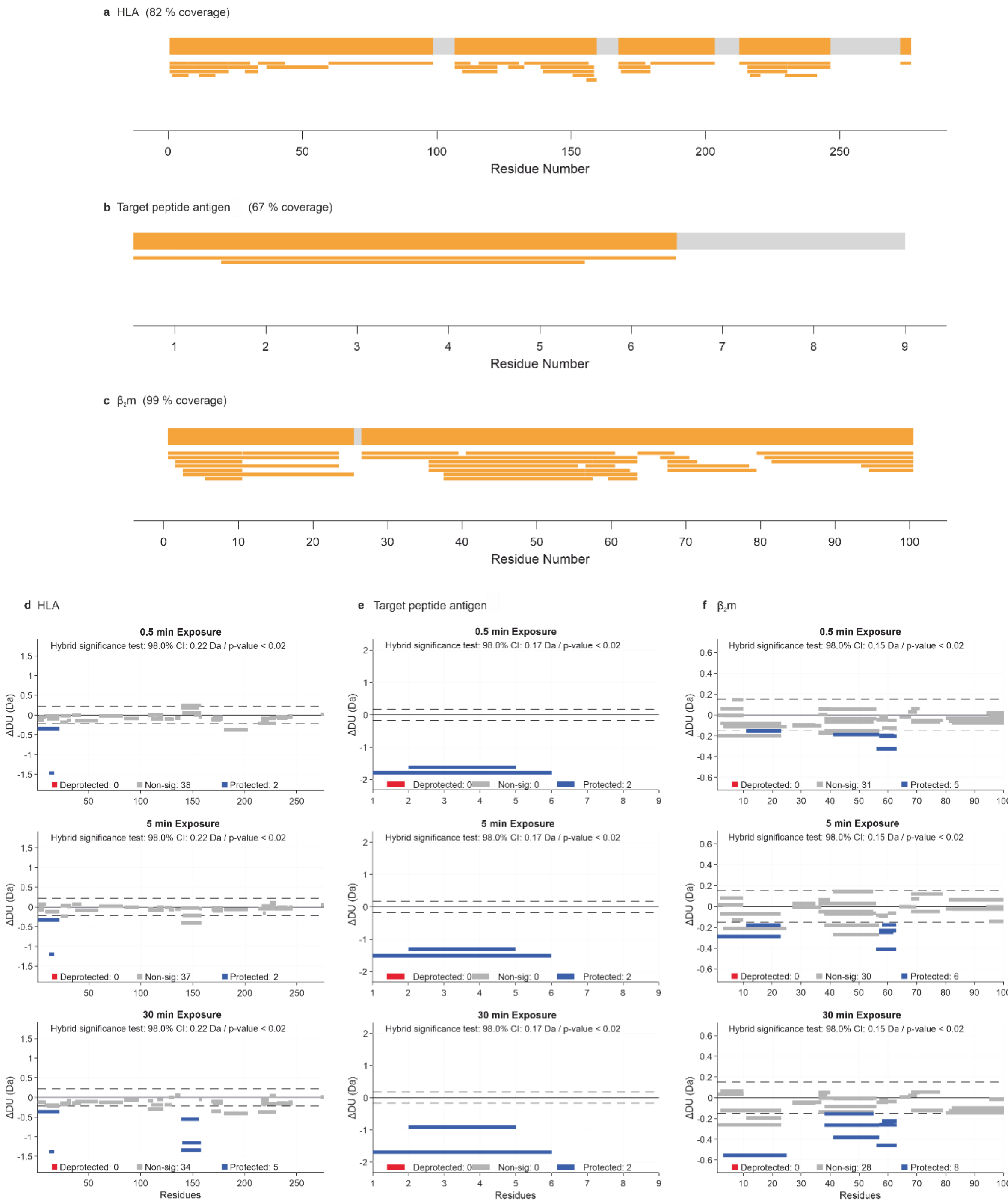

Supplementary Figure 5: HDX-MS of the TCR-C-pHLA complex. Sequence coverage map of **a** HLA, **b** target peptide antigen and **c** β2m in HDX-MS experiments for TCR-C. Woods' plot showing the differences in deuterium incorporation in each timepoints for **d** HLA, **e** target peptide antigen and **f** β2m domains. Peptides from the pHLA that were significantly protected and deprotected when incubated with deuterium in the presence of the TCR are shown in blue and red, respectively (hybrid statistical test, $p < 0.02$).

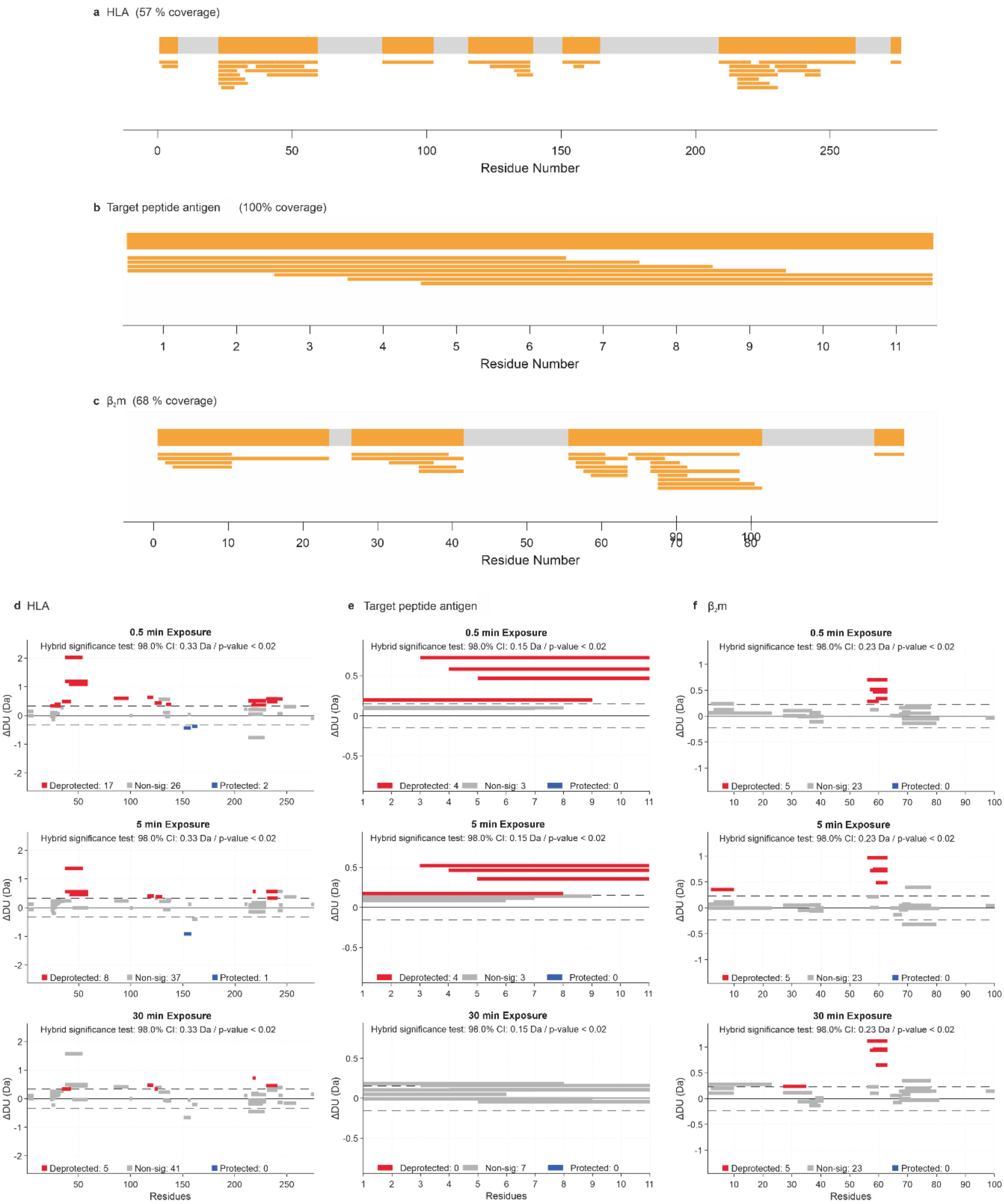

Supplementary Figure 6: HDX-MS of the TCR-E-pHLA complex. Sequence coverage map of **a** HLA, **b** target peptide antigen and **c** β2m in HDX-MS experiments for TCR-E. Woods' plot showing the differences in deuterium incorporation in each timepoints for **d** HLA, **e** target peptide antigen and **f** β2m domains. Peptides from the pHLA that were significantly protected and deprotected when incubated with deuterium in the presence of the TCR are shown in blue and red, respectively (hybrid statistical test, $p < 0.02$).

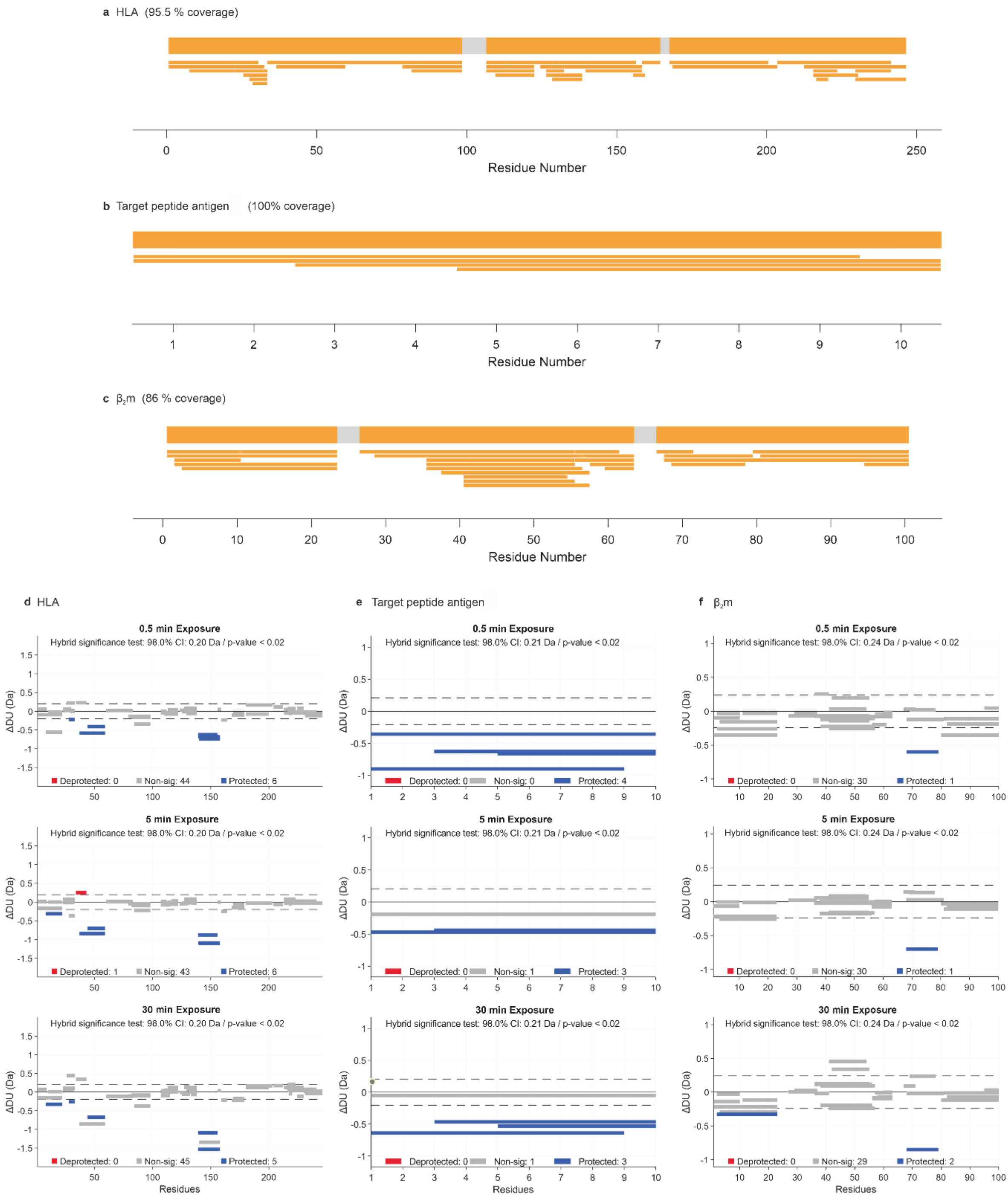

Supplementary Figure 7: HDX-MS of the TCR-D-pHLA complex. Sequence coverage map of **a** HLA, **b** target peptide antigen and **c** β2m in HDX-MS experiments for TCR-D. Woods' plot showing the differences in deuterium incorporation in each timepoints for **d** HLA, **e** target peptide antigen and **f** β2m domains. Peptides from the pHLA that were significantly protected and deprotected when incubated with deuterium in the presence of the TCR are shown in blue and red, respectively (hybrid statistical test, $p < 0.02$).

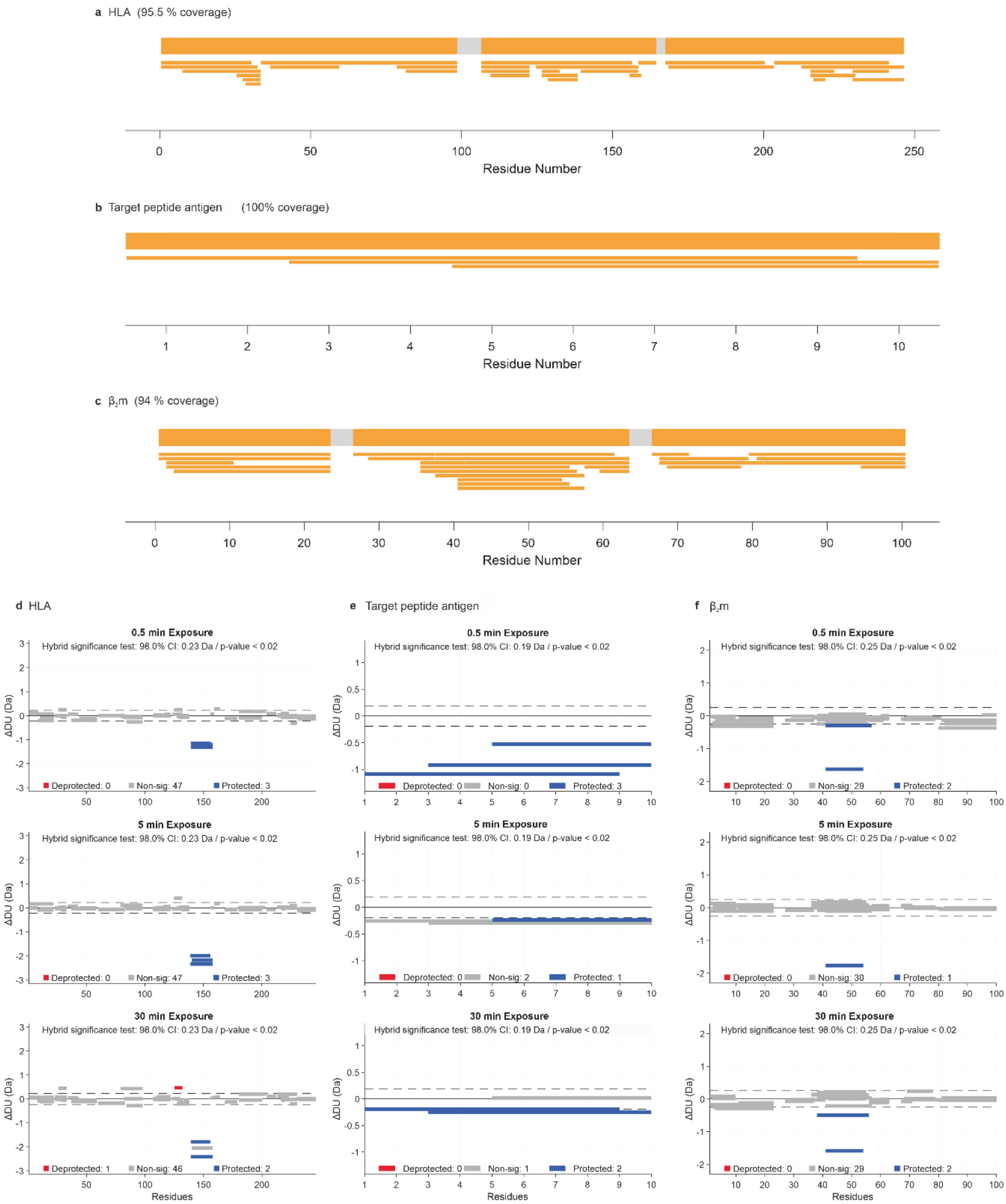

Supplementary Figure 8: HDX-MS of the TCR-F-pHLA complex. Sequence coverage map of **a** HLA, **b** target peptide antigen and **c** β2m in HDX-MS experiments for TCR-F. Woods' plot showing the differences in deuterium incorporation in each timepoints for **d** HLA, **e** target peptide antigen and **f** β2m domains. Peptides from the pHLA that were significantly protected and deprotected when incubated with deuterium in the presence of the TCR are shown in blue and red, respectively (hybrid statistical test, $p < 0.02$).

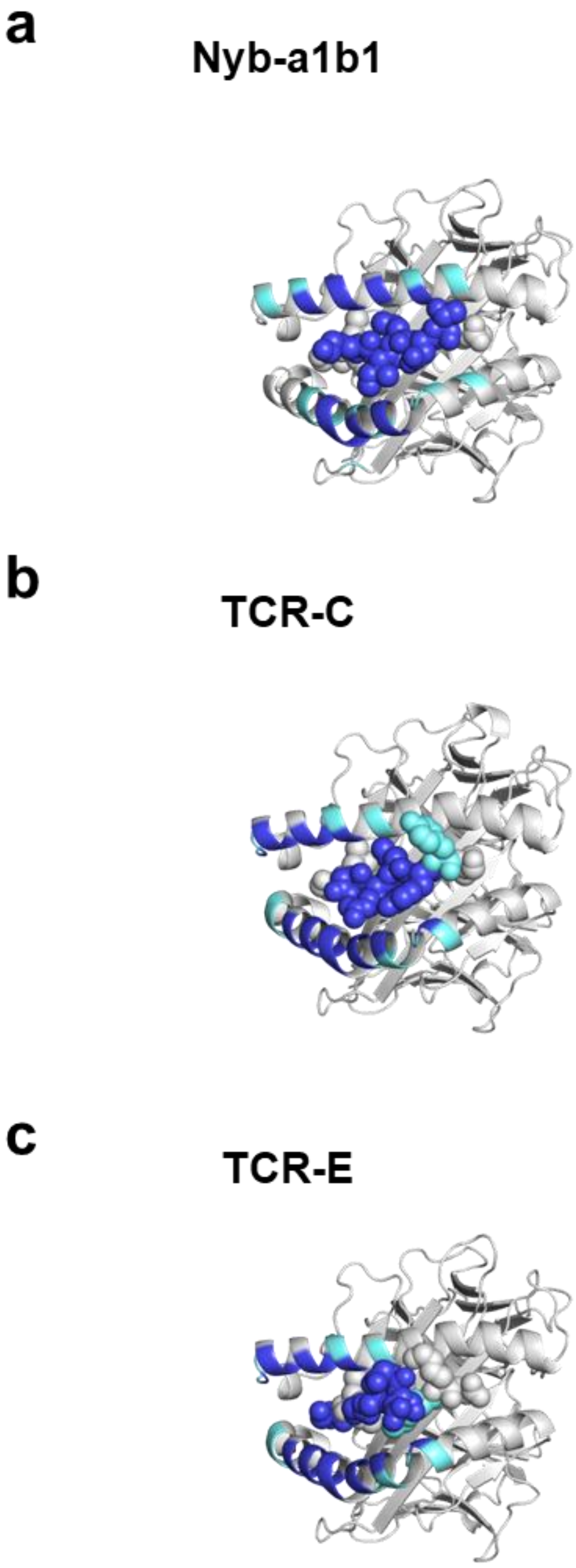

Supplementary Figure 9: Solvent accessibility changes in pHLAs upon TCR binding. Solvent accessibility values for each residue were calculated in the presence/absence of the TCR. Residues with decreased solvent accessibility in the presence of the TCR are coloured in light blue (< 50 % change in SASA) or dark blue (> 50 % change in SASA) for the (a) Nyb-a1b1, (b) TCR-C and (c) TCR-E complexes.

Supplementary Table 1. HDX Data Summary Table. SD = standard deviation, CI = confidence interval

Data Set	pHLA ± Nyb-awtb1	pHLA ± Nyb-a1b1	pHLA ± TCR-E	pHLA ± TCR-C	pHLA ± TCR-F	pHLA ± TCR-D
**HDX reaction details**	8 μM pHLA ± 10 μM TCR, phosphate buffered saline (Dulbecco), pD 7.0, 4 °C					
**HDX time course (min)**	0.5, 5, 30 min					
**HDX control samples**	Maximally-labelled controls were not performed					
**Back-exchange**	Not calculated and has been previously described for this system[1].					
**Sequence coverage**	pHLA: 72.5 %<br>Peptide: 100 %<br>β2m: 86 %	pHLA: 72.5 %<br>Peptide: 100 %<br>β2m: 86 %	pHLA: 68 %<br>Peptide: 100 %<br>β2m: 56.5 %	pHLA: 81.5 %<br>Peptide: 66.7 %<br>β2m: 99 %	pHLA: 94 %<br>Peptide: 100 %<br>β2m: 95.5 %	pHLA: 94 %<br>Peptide: 100 %<br>β2m: 95.5 %
**Number of peptides**	pHLA: 33<br>Peptide: 3<br>β2m: 20	pHLA: 33<br>Peptide: 3<br>β2m: 20	pHLA: 46<br>Peptide: 7<br>β2m: 28	pHLA: 40<br>Peptide: 2<br>β2m: 37	pHLA: 50<br>Peptide: 4<br>β2m: 32	pHLA: 50<br>Peptide: 4<br>β2m: 32
**Average peptide length / redundancy**	pHLA: 11.09 / 1.83<br>Peptide: 7.33 / 2.44<br>β2m: 12.30 / 2.86	pHLA: 11.09 / 1.83<br>Peptide: 7.33 / 2.44<br>β2m: 12.30 / 2.86	pHLA: 7.71 / 3.18<br>Peptide: 7.71 / 4.91<br>β2m: 9.22 / 2.72	pHLA: 11.55 / 2.05<br>Peptide: 5.00 / 1.67<br>β2m: 11.97 / 4.47	pHLA: 12.28 / 2.61<br>Peptide: 8.25 / 3.30<br>β2m: 13.55 / 4.47	pHLA: 12.28 / 2.61<br>Peptide: 8.25 / 3.30<br>β2m: 13.55 / 4.47
**Replicates (biological or technical)**	3 (technical)	3 (technical)	3 (technical)	3 (technical)	3 (technical)	3 (technical)
**Repeatability (average SD) (bound/apo)**	pHLA: 0.0366/ 0.0385<br>Peptide: 0.0360/ 0.0336<br>β2m: 0.0500/ 0.0559	pHLA: 0.0385/ 0.0395<br>Peptide: 0.0336/ 0.0214<br>β2m: 0.0559 / 0.0579	pHLA: 0.0578/ 0.0706<br>Peptide: 0.0393/ 0.0264<br>β2m: 0.0533/ 0.0990	pHLA: 0.0631/ 0.0512<br>Peptide: 0.0258/ 0.0371<br>β2m: 0.0581/ 0.0433	pHLA: 0.0662/ 0.0567<br>Peptide: 0.0494/ 0.0493<br>β2m: 0.0586/ 0.0674	pHLA: 0.0476/ 0.0567<br>Peptide: 0.0313/ 0.0493<br>β2m: 0.0544/ 0.0674
**Significant differences in HDX (Da)**	pHLA: 0.14 Da<br>Peptide: 0.17 Da<br>β2m: 0.22 Da<br><br>98 % Confidence, $p < 0.02$	pHLA: 0.15 Da<br>Peptide: 0.11 Da<br>β2m: 0.23 Da<br><br>98 % Confidence, $p < 0.02$	pHLA: 0.33 Da<br>Peptide: 0.15 Da<br>β2m: 0.23 Da<br><br>98 % Confidence, $p < 0.02$	pHLA: 0.22 Da<br>Peptide: 0.17 Da<br>β2m: 0.15 Da<br><br>98 % Confidence, $p < 0.02$	pHLA: 0.23 Da<br>Peptide: 0.19 Da<br>β2m: 0.25 Da<br><br>98 % Confidence, $p < 0.02$	pHLA: 0.20 Da<br>Peptide: 0.21 Da<br>β2m: 0.24 Da<br><br>98 % Confidence, $p < 0.02$

Supplementary References

1. T. Minshull, E. J. Byrd, M. Olejnik, A. N. Calabrese, J. Am. Chem. Soc. 2024, 146, 49, 33626–33639.
